# President’s Address to the Members of the Texas State Medical Association

**Published:** 1889-02

**Authors:** J. F. Y. Paine, F. E. Daniel

**Affiliations:** President; Executive Office, Texas State Med. Ass’n, Galveston, Texas; Secretary; Executive Office, Texas State Med. Ass’n, Galveston, Texas


### SOCIETY NOTES.

### PRESIDENT'S ADDRESS TO THE MEMBERS OF THE TEXAS STATE MEDICAL ASSOCIATION.

EXECUTIVE OFFICE, TEXAS STATE MED. ASS'N,
GALVESTON, TEXAS, January 22nd, 1889.

*To the Members of the Texas State Medical Association :*

It is my pleasing duty to remind you that the next Annual Session (twenty-first) of the Texas State Medical Association will be held in the City of San Antonio, from the 23rd to 26th of April, inclusive, 1889.

Eleven years ago the Society assembled in that goodly city, and doubtless all of those who were present thereat, retain pleasant recollections of the occasion. I applaud your selection of the place of meeting; for San Antonio contains much to please and interest. Beside the long scroll of history which lies behind her, she is a city of great commerical importance, possessing flourishing schools, handsome residences, well appointed hospitals, beautiful public grounds, and a population distinguished for refinement, culture and hospitality. These and matters material to the welfare of the Association invite your presence.

In addition to the usual scientific exercises, *action will be taken on the Report of the Committee on Revision of the Constitution and By-Laws*, and a full attendance is most earnestly requested, to the end that the important matter may be advantageously disposed of.

I have viewed with deep concern the apparent waning interest in the State Association, evinced by the annually decreasing attendance. Can it be possible that the various District Medical Societies in the State are accountable for this apathy, that they are rivals instead of complements of the Central Organization? I hope not. If in "Union and Harmony there is Strength," it follows that in segregation and discord there is weakness. Preserve the integrity of your local societies, but let them be as jewels in the diadem of the old Association. Let not small bodies, with diminished dignity and restricted usefulness, and full of jealousies and rivalries, take the place of your State Society; but lay aside unworthy aims and petty grievances, and band together in a common struggle against ignorance and imposture, and for advancement in useful knowledge—the noblest triumph attainable.

Let all respectable classes and interests in the profession come together, and by personal acquaintance, friendly intercourse, and mutual interchange of knowledge, personal differences and local prejudices be removed, the common stock of professional experiences increased, and the fraternity and usefulness of the profession promoted.

Let the State Association hold all of the socities in affiliation, and their membership in one grand harmonious organization, constantly increasing in numbers and winning fresh triumph over disease and suffering.

Let all members come and bring their professional associates; invite the young doctor just beginning his career, to attend the meeting; he will be introduced and made to feel at home among his friends. Encourage him to join the Society and begin to work in it.

I have the honor to be, with sincere interest,

Yonrs faithfully,

J. F. Y. PAINE, M. D.,

OFFICAL President Texas State Med. Ass'n.

F. E. DANIEL, M. D., Secretary.

